# Attention Increases the Temporal Precision of Conscious Perception: Verifying the Neural-ST^2^ Model

**DOI:** 10.1371/journal.pcbi.1000576

**Published:** 2009-11-26

**Authors:** Srivas Chennu, Patrick Craston, Brad Wyble, Howard Bowman

**Affiliations:** 1 Centre for Cognitive Neuroscience and Cognitive Systems, University of Kent, Canterbury, United Kingdom; 2 Department of Brain and Cognitive Sciences, Massachusetts Institute of Technology, Cambridge, Massachusetts, United States of America; University College London, United Kingdom

## Abstract

What role does attention play in ensuring the temporal precision of visual perception? Behavioural studies have investigated feature selection and binding in time using fleeting sequences of stimuli in the Rapid Serial Visual Presentation (RSVP) paradigm, and found that temporal accuracy is reduced when attentional control is diminished. To reduce the efficacy of attentional deployment, these studies have employed the Attentional Blink (AB) phenomenon. In this article, we use electroencephalography (EEG) to directly investigate the temporal dynamics of conscious perception. Specifically, employing a combination of experimental analysis and neural network modelling, we test the hypothesis that the availability of attention reduces temporal jitter in the latency between a target's visual onset and its consolidation into working memory. We perform time-frequency analysis on data from an AB study to compare the EEG trials underlying the P3 ERPs (Event-related Potential) evoked by targets seen outside vs. inside the AB time window. We find visual differences in phase-sorted ERPimages and statistical differences in the variance of the P3 phase distributions. These results argue for increased variation in the latency of conscious perception during the AB. This experimental analysis is complemented by a theoretical exploration of temporal attention and target processing. Using activation traces from the Neural-ST^2^ model, we generate *virtual ERPs* and *virtual ERPimages*. These are compared to their human counterparts to propose an explanation of how target consolidation in the context of the AB influences the temporal variability of selective attention. The AB provides us with a suitable phenomenon with which to investigate the interplay between attention and perception. The combination of experimental and theoretical elucidation in this article contributes to converging evidence for the notion that the AB reflects a reduction in the temporal acuity of selective attention and the timeliness of perception.

## Author Summary

Our visual system keeps pace with a rapidly changing stream of information as we view the natural world. To do so, it uses a strongly regulated system of attentional filters to constrain which visual stimuli are permitted to be fully processed to the level of conscious awareness. This article explores what happens when these filters are opened and closed in response to important visual stimuli. To understand these dynamics, our neural network model provides simulations of the role played by attention. These simulations can be tested by recording neural data in the form of ‘brain waves’ (EEG) and comparing the resultant signals to the output of the model. The data discussed here confirm a prediction of the model, which suggests that after the attentional filter has opened to allow one visual stimulus in, there is increased temporal variability or ‘jitter’ in the subsequent opening of the filter within an interval of about one-half of a second. These results have implications for the way our brains process multiple important stimuli perceived in rapid succession, such as the sequence of events that might occur at a critical moment in an airline cockpit or during an automobile accident.

## Introduction

During ongoing perception of the world, humans are constantly faced with an abundance of visual sensory information. As this information feeds through the various layers of visual cortex, it is progressively integrated by a sequence of cortical areas that gradually generalise over spatial information to extract complex structural detail [1]. Whereas early visual areas extract orientations, textures and borders, brain areas situated higher in the visual processing pathway can detect complex objects [2]. Bottom-up input flowing through this feedforward hierarchical pathway is constantly monitored for salience (e.g. task relevant or intrinsically prominent features like luminance or orientation pop-outs).

Within this general description of the visual system, attention is considered to play a key role, filtering out irrelevant information and selectively enhancing salient input for further processing. Here we investigate the temporal dynamics of visual attention with regard to its role in conscious perception, which becomes apparent when stimuli are presented in rapid succession [3],[4]. Such circumstances occur in rapid serial visual presentation (RSVP), in which stimuli are presented at a rate of approximately 10 items per second in the same spatial location. As each stimulus replaces its predecessor, its featural representation becomes fleeting due to masking effects, and a transient enhancement by attention is thought to be crucial in ensuring that salient items can be successfully encoded into working memory [5].

### The attentional blink

An apparent temporal limitation of visual perception is illustrated by the attentional blink (AB; [6]). The AB describes a finding that observers often fail to detect a second target stimulus (T2) presented in short succession (between 100 and 600 ms) after an identified first target stimulus (T1). If T2 is presented in immediate succession to T1, however, detection accuracy is typically excellent (‘lag 1 sparing’; [7]). Behaviourally, the AB has been replicated numerous times [8],[9]. It has also been investigated electrophysiologically [10], where researchers have compared grand average Event-related Potentials (ERPs) evoked by targets outside and inside the AB, to investigate how target processing differs during the AB.

Despite extensive study of the AB, its effect on the underlying temporal mechanisms of target identification remains to be fully explored. Evidence from ERP [10],[11] and priming [12],[13] studies suggest that targets, rather than being completely lost during the AB, are processed quite extensively, but fail to enter the final stage of conscious perception. Furthermore, researchers have found that when targets in RSVP consist of multiple features, observers often report features from items neighboring the target in the RSVP stream and make binding errors referred to as *illusory conjunctions* [14]. Behavioural analysis of the changes in the patterns of such binding errors provides strong support for the claim that the AB reveals a reduction in the temporal precision of the deployment of transient attention and target processing [15],[16].

### The ST^2^ model

In this article, we use the dynamics of temporal visual processing as embodied in the 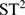 (Simultaneous-Type-Serial-Token) model, a connectionist model of temporal attention and working memory [5], to propose an explanation for the observed effect of the AB on the temporal precision of transient attention. The model explains a broad set of experimental findings relating to the AB, Repetition Blindness and RSVP in general. Before elaborating on our central hypothesis, we explain the fundamental principles of how the 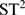 model describes temporal attention and working memory. For a more detailed description please refer to [5]. It should be emphasised that throughout this article, we retain the model's parameters as published in [17], and use it to generate predictions and virtual EEG traces comparable to human EEG data.

#### Types & tokens

The 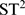 model employs a types-tokens account [18]–[20] to describe the process of working memory encoding. Types describe all feature related properties associated with an item. These include sensory properties, such as visual features (e.g. its shape, colour and the line segments comprising it) and also semantic attributes, such as a letter's position in the alphabet. A token, on the other hand, represents episodic information. It is specific to a particular occurrence of an item, and provides a notion of serial order. An item is encoded into working memory by creating a connection between a type and a token. At retrieval, tokens contain information about when an item occurred, in addition to a connection to a type. Thus the information stored in the tokens can be used to recollect both identity and temporal order of stimuli.

#### Model architecture

**Figure 1:**
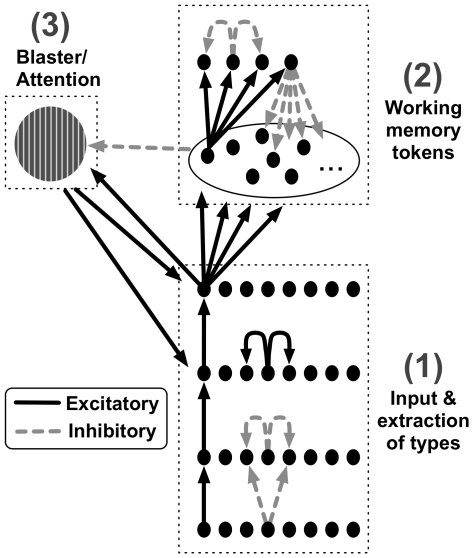
The ST^2^ model. (1) Input & extraction of types in stage one (2) Working memory tokens in stage two (3) Temporal attention from the blaster. Refer to [5] for an extensive description of individual layers, and the neural circuits comprising the nodes in each layer. Adapted from [17] with kind permission of MIT Press.

As illustrated in figure 1, the 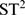 model can be divided into three parts. We describe them in turn:

- Input & extraction of types in stage one: Input values, which simulate target letters and digit distractors in the AB experiment, are fed into the model at the lowest layer of stage one. As activation propagates in a feed-forward manner, the following layers reflect masking in early visual processing and subsequent extraction of semantic representations. A task demand mechanism operates at the highest layer of stage one, and selects targets for encoding into working memory by suppressing the representations of distractors. Despite the fact that stimuli are presented serially during the AB task, processing within stage one may exceed the presentation time of sequentially presented items. Hence, these layers are parallel or simultaneous in nature, in that more than one node can be active at any one time.
- Working memory encoding in stage two: An item is encoded into working memory by connecting its type node in stage one to a working memory token in stage two. This process is referred to as ‘tokenization’. If at the end of a trial, the type node of a target has a valid connection to a token, the target is successfully ‘reported’, or ‘seen’, by the 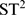 model. Inhibition between working memory tokens ensures only one token is active at any one time, thus enforcing a serialisation of working memory encoding.
- Temporal attention from the blaster: Temporal attention is implemented by a mechanism termed the *blaster*, which provides a non-specific excitatory input to nodes in the later layers of stage one in response to the detection of salient items (i.e., targets in the context of the AB). Transient Attentional Enhancement (TAE) from the blaster allows targets to become sufficiently active to initiate the tokenization process. During tokenization, the blaster is suppressed until encoding of the target has completed. The suppression prevents a second target from re-firing the blaster while the first target is being tokenised, thus preventing the episodic representations of the two targets from being conflated.

#### Virtual ERPs

Computational modelling of cognition is commonly focused on the replication of behavioural data. In particular, neural network models of cognition, in addition to replicating behavioural data, embody a hypothesis about underlying structure and function. On the empirical front, advances in technology now allow researchers to record ongoing brain activity correlated with a particular behaviour, such as the electrophysiological markers of neural processing. The natural question that arises is how such data can be combined with modelling to understand cognition at the neural level. This is possible because cognitive neural networks consist of nodes that derive from the functional characteristics of real neurons. Also, the activation of nodes in a model can be interpreted as the analogue of the activation of an assembly of real neurons. Consequently, activation traces in a neural model are comparable to aggregate neural activity expressed in EEG data.

The 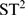 model simulates human behavioural accuracy in the AB. Using its neural network implementation, we generate virtual activation traces, called *Virtual ERPs* [17], by summing across layers of the model responsible for replicating specific cognitive functions. These traces are then compared to human ERPs across experimental conditions [17],[21]. The virtual ERP technique allows us to replicate, interpret and make predictions about human EEG data in a way similar to behavioural data. In particular, virtual ERPs allow us to validate our explanation of how the AB affects the temporal precision of conscious perception.

### Attentional precision, the AB and the ST^2^ model

The 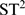 model suggests that working memory encoding involves creating a binding between the *type* of a stimulus (which can include its visual features and semantic attributes) and a *token* (an episodic representation specific to a particular occurrence of an item) [18],. In the 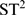 model, Transient Attentional Enhancement (TAE) from the blaster amplifies the *type* representation of a salient (i.e., task relevant) stimulus to assist in its binding to a token, in a process referred to as *tokenization*. This TAE can serve as an attentional gate, which can be temporarily deactivated to allow one target's encoding to be completed before a second is begun.

From the perspective of the 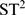 model, the AB is an artifact of the visual system attempting to assign unique tokens to targets [22]. More specifically, the process of encoding T1 into working memory is triggered by TAE, and TAE itself is subsequently suppressed until T1 encoding has completed. The period of TAE unavailability varies from trial to trial depending on how long it takes to tokenise T1, depending on its bottom-up strength. In an RSVP stream, if a T2 is presented 100–600 ms after a perceived T1 (as is the case during the AB), its processing outcome depends on multiple factors. T2's own strength determines its dependence on TAE, since highly salient T2s can ‘break-through’ the AB [31] and get encoded relatively early. T2s with strength values slightly lower in the range ‘outlive’ the AB (and thus the unavailability of TAE), and hence are indirectly influenced by T1 strength. Overall, the variability in the temporal dynamics of T2's encoding process is influenced both by T1 and T2 strengths. Hence, over all possible strengths, the 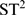 model proposes that there should be increased variance in processing latency for targets seen during the AB.

### Overview

This article investigates the hypothesis that diminished attentional control increases the *temporal jitter* in the latency of a target's working memory consolidation. The AB provides us with a suitable phenomenon with which to test our hypothesis: we propose that the reduced availability of attention during the AB increases the temporal noise in visual attention. To answer this question, we compare the EEG signatures evoked by targets seen outside vs. inside the AB, and determine whether there is a comparative increase in the variability of the latency of working memory encoding of targets presented inside the AB. EEG has the advantage of excellent temporal resolution, allowing us to study short-lived cognitive events that evoke changes in ongoing EEG activity. If one averages over multiple segments of such EEG activity time-locked to the event, the resulting averaged ERP waveform contains a number of positive and negative deflections, referred to as ERP components. To test for increased temporal jitter, we analyse the P3 ERP component, commonly associated with encoding items into working memory [10],[23].

However, analysis of averaged ERP components cannot directly inform our hypothesis. This is because the averaging collapses across and hence discards information about temporal fluctuations in the individual EEG trials contributing to the ERP. Given a set of trials that are averaged together, both decreases in amplitude and increases in latency variation within that set will attenuate the mean amplitude of the ERP. Hence, examining the average does not directly provide the necessary information to decide which of the two sources of variation in the individual trials (amplitude or latency) caused the reduction in ERP amplitude. Further, measures like 50% area latency analysis [24] cannot be used to measure latencies in single trials, due to the levels of irrelevant noise activity. Consequently, we employ time-frequency analysis techniques that provide alternative measures to investigate single trial dynamics underlying grand average ERPs. These methods enable us to perform a more fine-grained analysis of EEG data, and test our hypothesis using both qualitative and quantitative means.

In addition to presenting and analyzing human EEG data, we use the 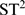 model's neural network implementation to generate *virtual P3* ERP components [17], which are hypothesised to correspond to the human P3 ERP component. For each of the experimental conditions, the virtual P3 is contrasted with the human P3, both at grand average and single trial level. This comparative evaluation allows us to validate the 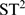 model and propose explanations for the human ERP effects.

## Results

The following section describes the human EEG activity evoked by targets outside and inside the AB. The data set used in the following analysis was the same as that contributing to the analyses presented in [17]. In the final part of the section, we use the 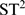 model to generate virtual ERPs, which we compare to human ERPs, and discuss the implications of this comparison for the theory underlying the 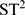 model. Please refer to the Materials and Methods section for more details on the experimental design and computational modelling.

### Analysis of behavioural accuracy

The experiment consisted of RSVP trials presented at a rate of 105.9 ms per item, with two letter targets, T1 and T2, embedded among digit distractors. T2 was presented at lags 1, 3 and 8 following the T1. The P3 EEG data analysed in this section was recorded at the Pz electrode. Please refer to the Materials and Methods section for further information.

Mean human accuracy for T1 identification was 82%. The accuracy of T2 identification (conditional on correct report of T1) was 83% at lag 1, 54% at lag 3, and 74% at lag 8. There was a significant effect of lag on accuracy (F(1.48,12.58)  = 15.58, MSE  = 0.03, p
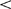
0.001, after applying a Greenhouse-Geisser correction on the degrees of freedom). Additionally, in pairwise comparisons, T2 accuracy was significantly lower at lag 3 compared to lag 8 (F(1,17)  = 11.66, MSE  = .03, p  = .003) and lag 1 (F(1,17)  = 60.88, MSE  = 0.01, p
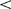
0.001). Consequently, the paradigm employed in this study evoked a reliable AB effect.

### Qualitative evidence for reduced temporal precision

**Figure 2:**
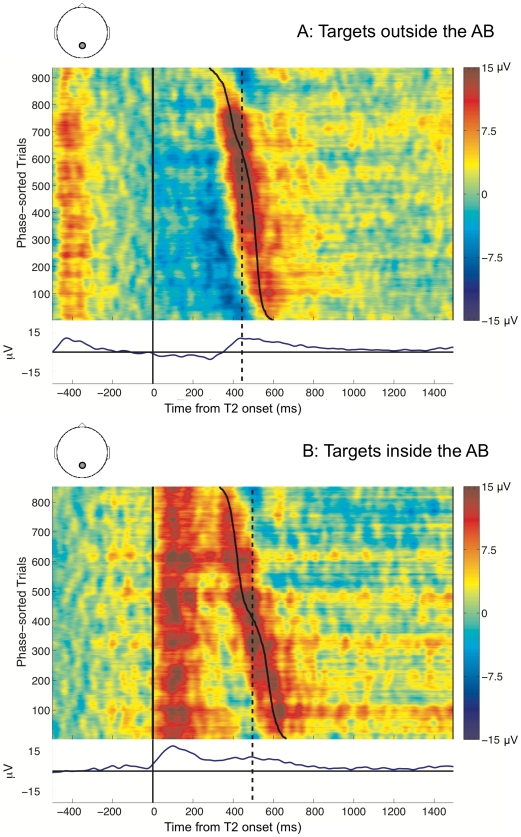
Human P3 ERPimages for targets seen outside and inside the AB. The ERPimages are time-locked to T2 presentation. Trials are sorted by phase at the peak latency of the grand average of the T2 P3 (indicated by the dashed line). The solid line illustrates the variation in phase, and is plotted by mapping the circular range of phase values onto the linear range of time-points encompassed by the wavelet.

The ERPimages [25] in figure 2 compare the P3 evoked by targets seen outside the AB (*seen T2s at lag 8 following a seen T1*) with targets seen inside the AB (*seen T2s at lag 3 following a seen T1*). They allowed us to visualise the EEG trials underlying the grand average P3 ERPs (plotted below them) for targets seen outside and inside the AB. These ERPimages represent time with respect to target onset along the X-axis (Note that trials are time-locked to T2 onset), individual trials along the Y-axis, and the single-trial EEG amplitude using a colour scale. The trials comprising these images were sorted from bottom to top by descending order of the phase angle of the single-trial P3 at the time point indicated by the dashed line, which was set to the peak latency of the corresponding grand average P3. This phase angle was estimated at the frequency at which the power of the P3 was maximal. This sorting method effectively ordered the trials according to the approximate latency of the single-trial P3 for a target, as estimated by a wavelet-based time-frequency analysis (see the Materials and Methods section for more details). The ERPimages were then plotted for each condition, with trials having longer latency P3s being placed at the bottom, and trials with shorter latency P3s at the top. Following from our hypothesis, for targets inside the AB, we expected to observe an increased “slope” in the red “smear” representing the P3. This would indicate that these targets suffer greater temporal variance compared to targets outside the AB.

A visual comparison of the ERPimages clearly suggested that the P3 for targets outside the AB (figure 2A) had relatively little variation in the phase angle across most trials. In other words, the P3 onset occurred at approximately the same time in these trials. In contrast, the P3 evoked by targets inside the AB (figure 2B) appeared to exhibit an increased temporal fluctuation, as reflected by the increased variance of the phase angle of the P3 across all trials. A natural consequence of this jitter in the temporal onset of the P3 was a ‘smearing out’ of the grand average ERP.

In summary, if there was indeed a reduction in the precision of the deployment of attention in response to targets during the AB, we expected this to indirectly affect the working memory encoding of targets as reflected by the P3. The ERPimages in figure 2 provided qualitative support for our hypothesis. We then extended this investigation by analysing the distribution of phase angles corresponding to the P3, to generate numerical evidence that could be verified statistically.

### Quantitative evidence for reduced temporal precision

To back up the qualitative comparisons of the previous section, we statistically analysed the time-frequency data obtained therein. We used an approach similar to inter-trial phase coherence analysis [25], but adapted the idea to directly examine the subject-wise P3 phase distributions and quantitatively compare temporal jitter. The phase angles used to sort the individual trials comprising the P3 ERPimages in the previous section formed a circular distribution [26] of angular data values that effectively represented the temporal latency between the onset of the target and its P3. By statistically comparing the variance in the distribution of phase angles across targets outside and inside the AB, we tested whether the visual differences observed were consistent across subjects.

To do so, we performed a subject-wise grouping of the P3 phase angles calculated at the peak latency of the grand average P3 for each condition (the same phase angles that were used to sort the ERPimages presented earlier). This generated multiple smaller distributions of P3 phase angles, one per condition and subject. These distributions were then modelled as von Mises distributions [26] for which the concentration parameter 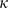 was calculated using maximum likelihood estimation. The 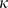 parameter of a distribution is a measure of its density around its mean value, and is an analogue of the inverse of its variance. The larger the 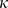 value of a circular distribution, the more concentrated it is around the mean. Importantly, 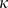 is a linear parameter, and can be compared using conventional statistical tools. Hence, in order to test whether targets inside the AB suffered from increased temporal jitter, we compared 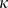 values of the subject-wise P3 phase distributions evoked by targets outside and inside the AB, using a standard one-way repeated-measures ANOVA. The results of the ANOVA validated what the visual differences observed in the ERPimages clearly indicated: The 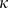 of the phase distribution for the P3 for targets outside the AB was statistically greater than that for targets inside the AB: Mean 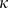 for targets outside the AB was 0.95, whereas mean 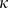 for targets inside the AB was 0.52 (F(1,17) = 15.21, MSE  = 0.11, p  = 0.001).

#### The potential confound of reduced amplitude

A potential confound in our time-frequency analysis arose from the well-established finding of reduced amplitude of the P3 for targets presented during the AB [10],[17],[27],[28]. Based on this finding, it could have been argued that the increased variation in the onset latency of the P3 for targets inside the AB was due to its reduced amplitude. This potential confound arose because the reduction in P3-related power could have effectively diminished the ability of the time-frequency analysis to calculate the phase of the P3. In other words, given a pair of P3 datasets, one with reduced P3 power compared to the other, the counter-argument to our interpretation would have claimed to explain the observed difference in P3 phase distributions by a reduction in P3 power during the AB.

To address this claim, we redid our statistical comparison of P3 phase angles, but with an additional step: before comparing the phase distributions, we first rejected trials from the *target outside the AB* condition with the highest power in the P3 window from 300–700 ms. This had the effect of reducing the mean power of the P3 for that condition, as it consisted only of the remaining trials. By performing this step, we discounted any influence of the amplitude of the P3 on the phase calculations. Indeed, we rejected a sufficiently large number of trials so as to reduce the mean P3 power for targets outside the AB to a value significantly smaller than that of the mean P3 power for targets inside the AB. Specifically, before trial rejection (i.e., including all 938 trials in the condition) the mean P3 power for targets outside the AB was 7.81 dB. This value was statistically greater than the mean P3 power for targets inside the AB: 6.64 dB (F(1,17)  = 33.07, MSE  = 0.37, p
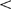
0.001). After rejecting 253 trials with the highest P3 power, the mean P3 power for targets outside the AB was reduced to 6.41 dB. This diminished power was statistically *lesser* than that for targets inside the AB (F(1,17)  = 5.76, MSE  = 0.086, p = 0.028). But in confirmation of our hypothesis, we found that the difference between the 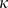 values for the phase distributions corresponding to the targets outside the AB (after trial rejection) and targets inside the AB conditions was still significantly different: Mean 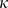 for targets outside the AB after trial rejection was 0.78; mean 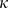 for targets inside the AB remained unchanged at 0.52 (F(1,17)  = 5.74, MSE  = 0.109, p = 0.028). Thus, this result addressed the potential confound. In other words, it confirmed that the differences observed in the P3 phase distributions reflected underlying differences in the corresponding temporal dynamics, which could not be explained away by differences in amplitude or power.

#### Phase distributions of the T1

**Figure 3:**
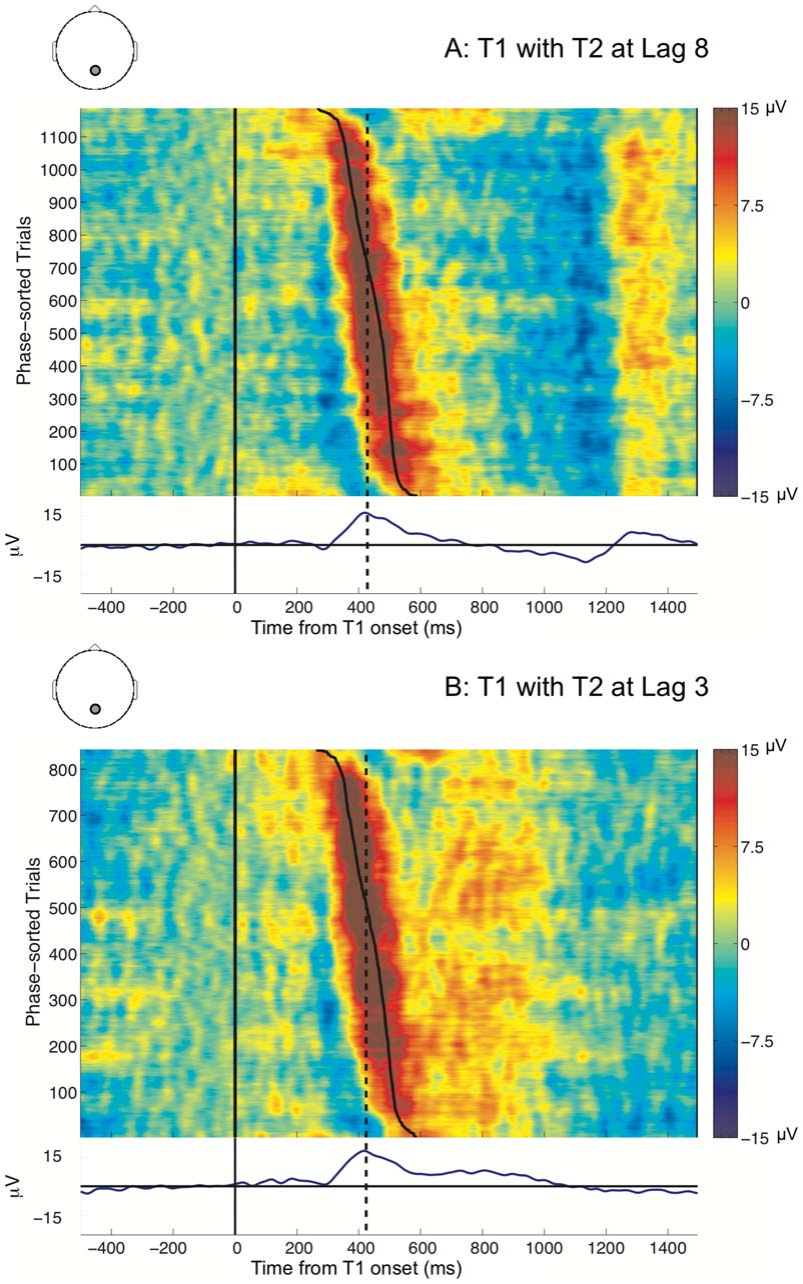
Human P3 ERPimages for seen T1s with T2 at lag 8 and at lag 3. The ERPimages are time-locked to T1 presentation. Trials are sorted by phase at the peak latency of the grand average of the T1 P3 (indicated by the dashed line). The solid line illustrates the variation in phase, and is plotted by mapping the circular range of phase values onto the linear range of time-points encompassed by the wavelet.

In order to further elucidate the statistical comparisons presented above, we compared the phase distributions for the T2s seen at lag 8 (outside) and lag 3 (inside) the AB with the phase distributions for the T1s preceding them. The ERPimages in figures 3A and 3B depict the P3s evoked by the seen T1s preceding seen T2s at lag 8 and lag 3, sorted by the phases at their grand average peaks at 428 ms and 424 ms, respectively (Note that these two conditions are equivalent to the target outside and target inside the AB conditions from figure 2, but are now time-locked to T1 onset and sorted by T1 phase).

In order to confirm the methodological validity of our time-frequency analysis, we checked for whether the proximity of the T1 and T2 P3s at lag 3 had adversely affected the estimation of phases. Specifically, it could have been that the preceding T1 P3 interfered with the wavelet analysis of the T2 P3 (despite the short wavelet length) and artificially increased the variance of its phase distribution. To test for this, we compared the T1 lag 8 P3 (figure 3A) and the T1 lag 3 P3 (figure 3B) conditions. If the wavelet analysis was indeed confounded, we expected a comparative increase in the variance of the phase distribution (and concomitant decrease in 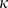) of the T1 lag 3 P3, mirroring the differences observed between the phase distributions of targets inside and outside the AB. But instead, we found that the T1 lag 3 P3 had a *higher* mean 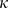 of 1.11 than the T1 lag 8 P3 with a mean 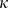 of 1.06, although this difference was not significant. (F(1,17) 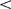
1, p
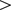
0.4). Thus, the T1 lag 3 P3 had a relatively high 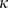 value despite its proximity to the T2 lag 3 P3. Overall, this suggested that the wavelet analysis was not confounded by this proximity, and was indeed capturing the EEG activity associated with the P3 being analysed.

The finding of increased temporal variance in T2 processing during the AB led us to the question of the influence of variance in T1 processing thereupon. Towards answering this question, we compared the differential effect of T1 on T2, across its presentation outside and inside the AB. We found that there were no visual differences apparent in the temporal variability of the T1 lag 8 P3 (figure 3A) and the T2 lag 8 P3 (figure 2A). In keeping with this observation, the 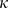 values of the corresponding phase distributions, 1.06 for the T1 lag 8 P3 and 0.95 for the T2 lag 8 P3, were not statistically distinguishable (F(1,17)  = 1.8, p = 0.2). In contrast, the visual comparison between the T1 lag 3 P3 (figure 3B) and the T2 lag 3 P3 (figure 2B) suggested that the former had higher temporal precision. Also, the 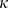 of the phase distribution for the T1 lag 3 P3 (mean 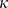 of 1.11) was statistically greater than that for the T2 lag 3 P3 (mean 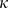 of 0.52): F(1,17)  = 15.34, MSE  = 0.202, p
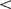
0.01. Taken together, these findings led to some important conclusions: Firstly, the jitter in T1 encoding was not affected by the lag position of the T2. Further, T1's influence on T2 jitter was temporally limited, i.e., T1 significantly increased T2 jitter *only* when T2 was presented within the AB window.

Following on from these findings, we were interested in whether there existed a direct relationship between the latencies of individual T1 and T2 P3s during the AB, as reflected by their phase values. To test this, we performed a trial-by-trial circular correlation of phase values of the T1 and T2 P3s at lag 3, but failed to find any relationship between the phases. This lack of an effect agreed with visual inferences from figure 2B, which suggested that sorting by the phase of the T2 lag 3 P3 did not result in any evident sorting of the T1 P3 preceding it. In the same vein, sorting by the phase of the T1 lag 3 P3 in figure 3B did not produce any sorting of the T2 P3 following it.

### Virtual ERPs from the ST^2^ model

In order to validate the 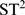 model, we used it to generate ‘artificial electrophysiological’ traces, so-called virtual ERPs [17]. In analogy to human ERP components, we generated virtual ERP components for targets outside and inside the AB. This approach, in addition to allowing us to validate the internal dynamics of the 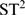 model, provided theoretical explanations for the human EEG effects observed in the previous section. Please refer to the Materials and Methods section for more details on how virtual ERPs and ERPimages were generated.

#### Simulated behavioural accuracy

The simulated behavioural accuracy from the 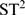 model was 85% for targets outside the AB and 31% for targets inside the AB. The 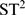 model thus qualitatively replicated the human behavioural data.

#### Virtual ERPimages

As with the analysis of human ERPs, grand average virtual ERPs are ‘blind’ to underlying trial-by-trial fluctuations, and could not be used to dissociate potential sources of aggregate effects. Hence, we investigated the correspondence between model and human ERP data at the level of individual trials. This was done by generating *virtual ERPimages* from the 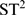 model. Similar to their human counterparts, virtual ERPimages illustrate the activation profiles of simulated trials making up a particular condition. However, human ERPimages unavoidably include inter-subject variability, occurring naturally in the neural dynamics across the subject pool. Hence, in order to generate comparable virtual ERPimages, we simulated inter-subject variability by introducing a small, random subject-wise delay in the processing of all stimuli in the model (see the Materials and Methods section for more details). For each such ‘simulated’ subject with a particular delay value, we executed a complete run of the model. This procedure was then repeated as many times as there were experimental subjects. In this way, we generated multiple datasets of simulated trials, one per subject and condition, which could then be statistically analysed. Further, by combining trials across all simulated subjects, we generated virtual ERPimages that captured some of the complexity present in the human ERPimages.

#### Qualitative comparisons to human ERPs

**Figure 4:**
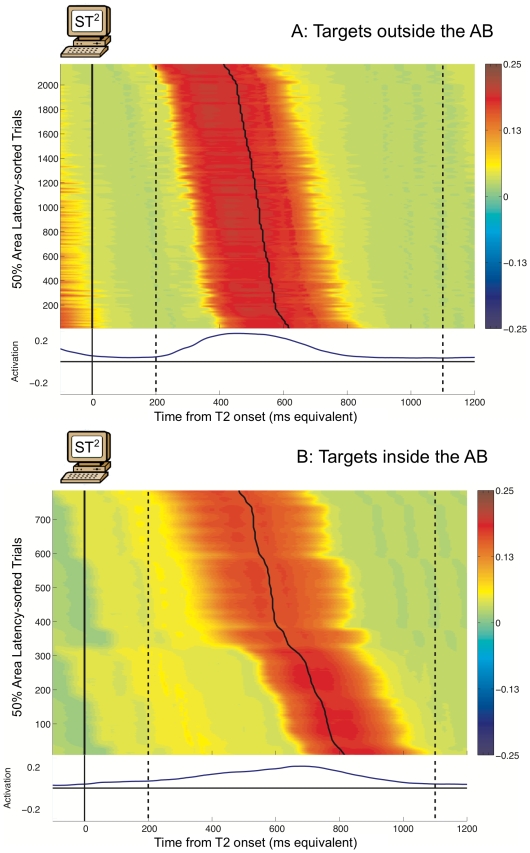
Virtual P3 ERPimages for targets seen outside and inside the AB. The ERPimages are time-locked to T2 presentation. Trials are sorted by 50% area latency (indicated by the solid line) within the window indicated by the dashed lines.

The virtual ERPimages in figure 4 depict the virtual P3s (with the corresponding grand averages plotted below them) evoked by targets seen outside the AB (*seen T2s at lag 8 following a seen T1*) with targets seen inside the AB (*seen T2s at lag 3 following a seen T1*). These conditions mirror the ones analysed with the human EEG data. However, note that in contrast to the human ERPimages in figure 2, the virtual ERPimages only depict simulated activity related to the target in question. This was done for visual clarity, and was possible because we could isolate and selectively plot activation generated by a specific target in the 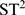 model. Further, the trials comprising the virtual ERPimages were sorted by 50% area latency [24] of the appropriate virtual P3 within 200–1100 ms after target onset (indicated by dashed lines in figure 4) in each trial. We used the 50% area latency measure with virtual ERPs, since, unlike human ERPs, they were free from noise.

The ERPimages for the virtual P3 showed that the simulated EEG activity for targets outside the AB (figure 4A) was well aligned with target onset. In comparison, the virtual P3 for targets inside the AB (figure 4B) varied over a wider range of latencies. This difference provided a qualitative replication of the pattern of effects in the human P3 ERPimage (figure 2).

#### Quantitative comparisons to human ERPs

We statistically tested the observed qualitative differences in the virtual ERPimages, by comparing across simulated subjects the 50% area latencies of the virtual P3 within the 200–1100 ms window (the same latency values used to sort the virtual ERPimages). We found that targets outside the AB (mean latency  = 529.68 ms) had significantly earlier mean 50% area latency than targets inside the AB (mean latency  = 649.31 ms): F(1,17) 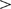
100, MSE  = 6.69, p
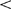
0.001. This shift in the mean latency of the virtual P3 during the AB follows from the delayed consolidation hypothesis of the 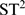 model [29]. Previous ERP studies of the AB [27],[30] have reported such a shift in the human P3, which is mirrored by the observed shift in the peak latency of the grand average human P3s in our data.

To quantitatively test whether the virtual P3 suffered from increased temporal variance for targets inside the AB, we compared the standard deviations of the 50% area latencies of the virtual P3 across simulated subjects. In keeping with the qualitative differences observed in the virtual ERPimages, virtual P3s evoked by targets outside the AB had much smaller standard deviation in their latencies (mean S.D. = 44.80 ms) than those evoked by targets inside the AB (mean S.D. = 93.81 ms). This difference was highly significant: F(1,17) 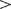
100, MSE  = 0.052, p
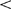
0.001, and mirrored the statistical differences in the phase distributions of the human P3s.

## Discussion

Our qualitative and quantitative comparisons of human ERPimages support the notion of increased temporal variance in target processing during the AB. Further, we have shown that the observed differences in the phase distributions of targets seen outside and inside the AB are indeed real, and cannot be explained by differences in amplitude or any methodological limitations. Finally, our analysis also suggests that T1 processing significantly influences the variance in T2 processing during the AB window, though this could not be confirmed by a trial-by-trial correlation of T1 and T2 phases. At the end of this section, we interpret this finding in relation to predictions from the 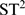 model.

The virtual ERPs and ERPimages have provided a means for visualising the theory underlying the 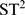 model, at a fine-grained level of detail. Using this novel methodology of comparing model and data both at the level of averages and single-trials, we have shown that, in line with the 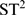 model's hypothesis, activation traces of attentional response and consequent working memory encoding show decreased temporal precision for targets inside the AB compared to targets outside it. However, it is clear from the virtual ERPimages that the virtual P3 for targets inside the AB is exaggerated in terms of its delay and duration. This is a consequence of the strong suppression of TAE in the 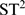 model during target consolidation. But it does not affect the qualitative comparisons with the human ERPimages, or the conclusions we have drawn therefrom.

To further clarify the causes of temporal variability in the 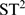 model, we now summarise the underlying mechanisms that produce it. In the model, transient attentional enhancement (TAE) is evoked by detection of a target, and this attention triggers the encoding of that target into working memory by binding its type representation to a working memory token, which results in this target being correctly reported at the end of the trial. For targets presented outside the AB, such as a T2 at lag 8, the TAE mechanism (i.e. the blaster circuit) is readily available. It fires as soon as an item is classified as a target, and encoding is thus tightly timelocked to the target onset. Thus, there is little variation in the tokenization delay and consequently the latency of the virtual P3. Also, because attention is immediately deployed, the model's behavioural accuracy at detecting targets outside the AB is high.

However, as described previously, the processing of a target presented during the AB (a T2) is complicated by multiple factors. Firstly, T1's strength determines the period of unavailability of the blaster. In addition, T2's own strength determines its dependence on the blaster, as highly salient T2s (at upper end of the range of target strength) can break-through the AB [31] and get encoded early. T2s with slightly lower strength values can outlive the AB, but require the blaster's enhancement. Quite a few T2s, however, have insufficient strength to survive the delay in the blaster response and are missed, producing an AB. This complex relationship between T1 and T2 at lag 3 increases temporal variability in the latency of T2's virtual P3, but implies that the model does not predict a strong, direct correlation between T1 and T2 P3 latencies. A possible reason for the lack of any such correlation between the corresponding human P3 phase distributions could be insufficient variation in T1 strength in our experiment, combined with noise obscuring a weak effect. With sufficient variation in T1 strength (for example, when comparing across T1 masked vs. unmasked) the dynamics of the 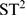 model propose a stronger relationship between the *duration* of the T1 P3 and the latency of the T2 P3 during the AB. Indeed, the model suggests that there should be a reciprocal influence of T1 strength on its encoding duration [29], which would in turn have implications for T2 P3 latency. Testing for such a relationship would be informative, but a detailed investigation of this topic is beyond the scope of this article.

### Related work

Our experimental results and theoretical explorations complement and inform previous research on temporal selection and the AB. We now discuss these findings and propose interpretations in terms of the 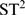 model.

#### Chun (1997), Popple and Levi (2007)

Chun [32] provided initial evidence regarding the effect of the AB on temporal binding. Employing an RSVP paradigm consisting of letters enclosed in coloured boxes and target letters marked by a distinctively coloured box, he investigated the distribution of responses made by participants when either one or two targets were presented. He calculated the centre of mass of this distribution for targets outside and inside the AB, and found that for targets outside the AB, the distribution was roughly symmetrical around the target position. But for targets inside the AB, he observed a significant shift in the response distribution toward items presented after the target. In addition, behavioural data presented in [32] shows that the variance of the response distribution for T2 report increases when it is presented inside the AB. Popple and Levi [15] presented additional behavioural evidence consistent with Chun's findings [32]. Using a colour-marked RSVP paradigm where each item had two features (colour and identity), they found that incorrect responses mostly came from the distractor items that were presented close to, and generally following the T2. In addition, they observed that this distribution of responses for T2 showed a pronounced increase in its spread compared to T1.

These findings are well explained by the 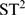 model. In 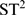, the inhibition of the blaster delays the deployment of attention to a T2 presented during the AB. Consequently, non-targets presented right after the T2 are more likely to be tokenised when the second stage becomes available, resulting in the observed shift in the response distribution. Also, as explained in the previous section, due to a combination of factors influenced by T1 and T2 strengths, there is increased temporal variability in T2's encoding process. This in turn leads to increased variation in the behavioural response for T2s presented inside the AB.

#### Vul, Nieuwenstein and Kanwisher (2008)

Vul, Nieuwenstein and Kanwisher [16] propose that temporal selection is modulated along multiple dimensions by the AB. They employed an RSVP paradigm consisting of letters, with targets delineated by simultaneously presented annular cues. Their behavioural analysis suggests that target selection is affected by the AB in one or more of three externally dissociable dimensions discussed below: suppression, delay, diffusion. However, with the 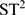 model, we demonstrate that all three can result from the suppression of attention.

- *Suppression* refers to the reduction in the effectiveness of temporal selection during the AB, and a concomitant increase in random guesses. Vul et al. [16] measured this effect in the form of a decrease in the mean probability of selecting a proximal response (from 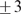 item positions) around the target, when it occurs during the AB. In contrast to results in [15], they found a significant decrease in this value for T2s during the AB. In the 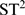 model, suppression can be explained by a reduction in the probability of a target triggering the blaster. During the AB, a relatively large percentage of T2s fail to fire the blaster and do not have enough bottom-up strength to be tokenised. The model would hence predict the suppression observed by [16], because the percentage of trials in which the blaster fires in response to a T2 would be reduced during the AB. Furthermore, as participants were forced to indicate a response for both targets [16], this reduction would translate to an increase in the number of random guesses for the T2. Finally, as one would expect, the time course of suppression follows the time course of the AB as simulated by the 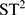 model.
- *Delay* refers to a systematic post-target shift in the locus of responses chosen for T2 when compared to T1. Vul et al. [16] quantified delay as the centre of mass of the distribution of responses for each target, calculated similarly to the API (Average Position of Intrusions) measure in [14] and the intrusion index score in [32]. This notion of an increase in the latency of attentional selection is reflected in the 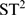 model. Specifically, suppression of the blaster during T1 encoding results in an increase in the latency of its response to a T2 during the AB (see [29] for more details on delayed T2 consolidation in the 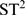 model). As a result, in an RSVP paradigm like that used by [16], items presented after T2 are more likely to get the benefit of the blaster and get chosen as responses, resulting in the observed shift in the response distribution. However, this shift in the locus of responses observed by [16] seems to persist at late T2 lag positions well beyond the duration of the AB, and is somewhat more puzzling. This finding could perhaps be attributed to the cognitive load associated with holding T1 in working memory.
- *Diffusion* refers to a decrease in the precision of temporal selection, corresponding to an increase in the overall spread in the distribution of responses during the AB. Vul et al. [16] estimated diffusion by comparing the variance around the centre of mass of the response distributions for T1 and T2, and found that it is significantly increased for T2s during the AB. This observation is explained by the 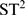 model as follows: in the context of the paradigm in [16], there would be increased temporal variation in T2 encoding because of the influence of T1 processing. Hence, due to the influence of both T1 and T2 strengths on response selection, erroneous responses further away from the target position would get selected for tokenization, producing increased variance in the distribution of responses. Again, the time course of diffusion is similar to that of suppression, and is in keeping with the window of the AB predicted by the 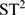 model.

In summary, we think that a single underlying mechanism of variation in the temporal dynamics of attention from trial to trial could potentially explain the three effects observed in [16]. An explicit computational account of these three dimensions in terms of the 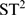 model is beyond the scope of this article (and would require it to be extended to simulate the conjunction of multiple stimulus features). Nevertheless, the explanation proposed above highlights the role that the temporal dynamics of transient attention would play in explaining these effects.

#### Sergent, Baillet and Dehaene (2005)

Sergent, Baillet and Dehaene [33] combined behaviour and EEG to investigate the timing of brain events underlying access to consciousness during the AB. They analysed early and late ERP components evoked by a pair of targets, a T1 followed by a T2 either at a short lag (equivalent to our *inside the AB* condition) or at a long lag (equivalent to our *outside the AB* condition). They plotted unsorted ERPimages to visualise the inter-trial variation in the EEG activity, and found that when T2 was presented within the AB, T1's P3 influenced the temporal dynamics of the ERP components correlated with conscious access to T2. In particular, the ERPimage depicting their T1 and T2 P3s clearly shows that even when T2 is seen during the AB window, it evokes a more ‘smeared out’ P3 as compared to the T1. However, the analysis of single-trial data in [33] presents ERPimages that are not sorted (unlike the phase sorting we have performed in this article), thus limiting their interpretation. Further, they did not compare temporal variability of targets seen outside and inside the AB. Despite these differences, their data agree well with ours, and provide qualitative support for our hypothesis of reduced temporal precision during the AB. This is because we would expect increased inter-trial variability in the P3 evoked by a T2 inside the AB to result in a ‘smearing out’ effect in its ERPimage, when trials are plotted after smoothing, but without sorting by phase.

### Conclusion

In this article, we have presented human ERP evidence in favour of a reduction in the temporal precision of transient attention during the AB. The AB provides us with a suitable phenomenon with which to investigate the interplay between attention and perception. The interplay between these tightly linked cognitive processes is adversely affected during the AB, producing the reduction in precision observed in behavioural and EEG data.

Using ERPimages, we have provided qualitative evidence arguing for an increase in temporal variation in the dynamics of P3s evoked by targets seen outside vs. inside the AB window. This evidence is supported quantitatively, by statistical comparison of the phase distributions corresponding to the P3. This analysis suggests that there is significantly increased temporal jitter in the ERP activity evoked by targets inside the AB. This notion of a decrease in the temporal precision of attention is inherent in the theoretical framework of the 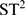 model. Specifically, we have used the 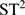 model’s neural implementation to generate both virtual ERPs and ERPimages, which we have then compared to their human counterparts. We believe that correlating model and electrophysiological data in this way provides a two-fold benefit. Firstly, it has provided a sufficient explanation for the modulatory effects of the AB on the temporal precision of visual processing. Secondly, it has allowed us to instantiate and test the model at the level of single-trial dynamics, and show that the theoretical assumptions about the nature of temporal visual processing embodied by it can be validated using EEG data, in addition to traditional behavioural verification. We believe that the combination of experimental and theoretical analysis presented in this article contributes to converging evidence for the notion that the AB results in a reduction in the temporal acuity of selective attention, which is an important mechanism for ensuring the timeliness of conscious perception.

## Materials and Methods

### Experimental methods

This section describes the experiment (the same as Experiment 2 from [17]) used to collect the human EEG data analysed in this article.

#### Participants

We recruited 20 under- and postgraduate university students (mean age 23.1, SD 3.2; 10 female; 18 right-handed). Two participants were excluded from the analysis. The first one seemed to be a non-blinker [34], as his performance was at ceiling across all three lags. The second participant was excluded due to persistently high oscillations in the alpha band throughout the experiment. Hence, 18 participants remained for behavioural and EEG analysis (mean age 22.5, SD 2.7; 9 female; 18 right-handed). Participants were free from neurological disorders and had normal or corrected-to-normal vision.

#### Ethics statement

All participants provided written consent and received 10 GBP for participation. The study was approved by the ethics committee of the Science, Technology and Medical Studies Faculty at the University of Kent.

#### Stimuli and apparatus

We presented alphanumeric characters in black on a white background at a distance of 100 cm on a 21” CRT computer screen (1024×768@85 Hz) using the Psychophysics toolbox [35] running on Matlab 6.5 under Microsoft Windows XP. Stimuli were in Arial font and had an average size of 1.03°×0.69° visual angle.

#### Procedure

**Figure 5:**
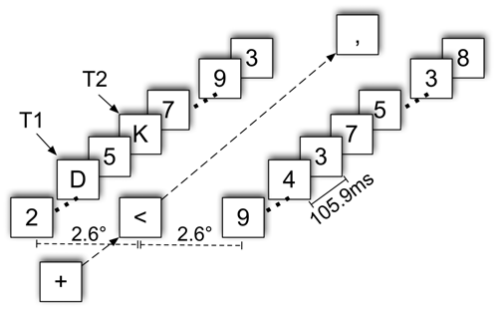
Experimental design. Each trial began with a central fixation cross, which turned into an arrow indicating the side on which targets would be presented. This was followed by two simultaneous RSVP streams on either side of fixation. The central arrow was finally replaced by a dot or a comma.

Participants viewed 4 blocks of 100 trials. Before starting the experiment, participants were asked to make 5 eye blinks and 5 horizontal eye movements to record the typical pattern of EOG activity. This was used to configure the algorithm for eye blink artifact rejection. Participants performed 8 practice trials, which were not included in the analysis. As shown in figure 5, RSVP streams were preceded by a fixation cross in the centre of the screen. After 400 ms, the cross turned into an arrow indicating the side on which the targets would be presented. After 200 ms, two streams of digits were simultaneously presented at an equal distance of 2.6° visual angle to the left and right of fixation. The RSVP stream consisted of 35 items presented for 105.9 ms each with no inter-stimulus interval. For 84% of trials in a block, the stream on the side indicated by the arrow contained 2 targets (T1 & T2), in 16% of trials both streams were made up of distractor digits only. The ‘distractor only’ trials were randomly inserted to make the occurrence of targets less predictable. In a trial, T1 and T2 were selected from a list of 14 possible targets (A, B, C, D, E, F, G, H, J, K, L, N, P, R, T, U, V, Y); distractors could be any digit except ‘1’ or ‘0’. T1 appeared between stream position 5 and 17; T2 followed T1 at position 1 relative to T1 (no intervening distractors - lag 1), position 3 relative to T1 (2 intervening distractors - lag 3) or position 8 relative to T1 (7 intervening distractors - lag 8). The arrow remained in the centre of the screen until the streams were over and then turned into either a dot or a comma.

Before the experiment started, participants were told to keep their eyes fixated on the centre of the screen from presentation of the cross until the dot/comma, as trials with eye movements would be identified in the EOG and excluded from the analysis. Participants were told to direct their covert attention towards the indicated stream, search for the two target letters and remember whether the last item was a dot or a comma. Participants were informed that streams could contain either two or zero targets. Following stream presentation, participants were presented with the message ‘If you saw letters - type them in order, then dot or comma for the final item’ and entered their response without time pressure using a computer keyboard. The dot-comma task was included to ensure that participants kept their eyes fixated on the centre of the screen throughout the RSVP stream.

#### EEG recording

EEG activity was recorded from Ag/Ag-Cl electrodes mounted on an electrode cap (FMS, Munich, Germany) using a high input impedance amplifier (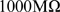, BrainProducts, Munich, Germany) with a 22-bit analog-to-digital converter. Electrode impedance was reduced to less than 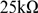 before data acquisition [36]. EEG amplifier and electrodes employed actiShield technology (BrainProducts, Munich, Germany) for noise and artifact reduction.

The sampling rate was 1000 Hz and the data was filtered at 80 Hz low-pass and 0.25 Hz high-pass at recording. 20 electrodes were placed at the following standard locations according to the international 10/20 system [37]: Fp1, Fp2, Fz, F3, F4, F7, F8, Cz, C3, C4, C7, C8, Pz, P3, P4, P7, P8, Oz, O1, O2, T7 and T8. Horizontal eye movements, recorded from a bipolar EOG channel placed below and to the left of the participant's left eye, indicated when participants had moved their eyes away from fixation and towards one of the RSVP streams.

#### EEG analysis

EEG data was analysed using BrainVision Analyzer (BrainProducts, Munich, Germany), in conjunction with EEGLAB 6.01b [25] and custom MATLAB scripts. In Analyzer, the data was first referenced to a common average online and re-referenced to linked earlobes offline. Left mastoid acted as ground. Signal deviations in the EOG channel of more than 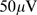 within an interval of 100 ms were identified as eye blink and movement artifacts, and a window of 200 ms before and after an artifact were marked for rejection. To verify that these artifacts were accurately identified by the algorithm, we performed a manual inspection after the algorithm had been applied.

The continuous EEG data from each participant was loaded into MATLAB and low-pass filtered at 25 Hz. The data was then segmented into trials. This was done by extracting a time window of −500 ms to 1500 ms around the target onset times for the conditions of interest, namely seen T2s at lag 8 following a seen T1 (targets outside the AB), seen T2s at lag 3 following a seen T1 (targets inside the AB), seen T1s with a seen T2 at lag 8 (T1 lag 8) and seen T1s with a seen T2 at lag 3 (T1 lag 3). Trials marked as containing artifacts by the procedure defined above were excluded from further analysis. After artifact rejection, the total number of trials in the above conditions were - seen T2s at lag 8: 938; seen T2s at lag 3: 853; seen T1s with T2 at lag 8: 1188; seen T1s with T2 at lag 3: 843. A linear detrend function was applied to all trials. Finally, trials in all conditions except the targets inside the AB condition were baselined to the −200 ms to 0 ms window before presentation of the target to which the trial was time-locked. Trials in the targets inside the AB condition were baselined to the −500 to −300 ms window before T2 presentation, to ensure that there was no T1-related activity in the baseline window.

To plot grand average ERPs, ERPimages and to perform statistical analyses, the P3 was generated using data recorded at the Pz electrode. Repeated measures Analysis of Variance (ANOVA) were performed on subject averages using custom MATLAB scripts, which implement functions from [38]. Where appropriate, p-values were adjusted using Greenhouse-Geisser correction.

#### ERPimages

To plot the ERPimages in this article, trials were vertically sorted by the phase angle, which is calculated using wavelet-based time-frequency analysis, performed separately for each trial. A half-cycle Morlet wavelet (cycle length of 0.5) at 1.53 Hz was used as the template for the time-frequency analysis. This frequency was set by identifying the peak power spectral density of the P3 EEG trials contributing to the outside the AB condition. This frequency generated a wavelet with a half-cycle duration of 326.8 ms, which when centred on the peak of the grand average P3, ensured that it only captured activity related to the single-trial P3 evoked by the target in question. The time-frequency analysis returned a pair of two-dimensional matrices, indexed by trial number and time point, and consisting of the power and phase values calculated at the specified frequency. In order to then pick out the phase of the phasic P3 across trials in a condition, the peak of the grand average P3 ERP for that condition was used. This peak occurred at 444 ms for the T2 at lag 8, and at 496 ms for the T2 at lag 3. For each of these experimental conditions, this then produced a circular distribution [26] of phase values within the range 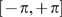 radians, consisting only of the phase values at the chosen time point, across all the trials for the condition. Finally, ERPimages were plotted as a colour-map for each condition by sorting the trials based on these phase values, and then vertically smoothing over them using a sliding window of 50 trials, in order to better visualise patterns in the single trials underlying the grand average ERP. The phase distributions thus generated for P3s outside and inside the AB were statistically compared using R [39] and MATLAB, by first grouping the phase values by subject and estimating the concentration parameter 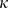 for the subject-wise phase distributions using maximum likelihood estimation, and feeding them into a standard one-way repeated measures ANOVA.

### Computational methods

#### ST^2^ model configuration

The input patterns presented to the model were comprised of 25 items presented for 20 timesteps (equivalent to 100 ms) each. T1 appeared at position 7 in the RSVP stream and T2 followed T1 with 0 to 7 distractors (lags 1–8) between the two targets. All target and distractor strength values and model parameters are fixed to be the same as those published in [17].

#### Virtual ERPs and ERPimages

The philosophy we adopted to generate virtual ERPs from activation dynamics of the model (discussed in greater depth in [17]), was to employ a straightforward approach with no parameter fitting, while keeping it as close as possible to the processes assumed to occur in the brain (For a more neurophysiologically detailed account of ERP generation, see [40]). Cortical pyramidal neurons in the brain have inter-layer connectivity and are aligned perpendicular to the cortex, which is why they are thought to be a major contributor to human EEG [41]. We assumed the major weighted connections across layers in the 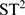 model (figure 1) to be analogues of synaptic projections in the brain. Accordingly, we generated virtual ERPs by summing across the excitatory postsynaptic potentials of all relevant nodes in the layers that perform cognitive functions reflected by the human ERPs in question. These virtual ERPs are averages of the activation profiles collapsed across all trials in a complete simulation run of the model, encompassing a range of target strengths. It is obvious, however, that virtual ERPs thus calculated remain a coarse approximation of human ERPs, as physical topography of the brain, skull and scalp are not taken into account. Consequently, though one can realistically only expect to obtain a qualitative match to human ERPs, virtual ERPs can serve as valuable predictors of the patterns of change in human ERPs, particularly with regard to temporal factors.

#### Virtual P3

The human P3 is a broad component spread over a large group of parietally centred electrode sites, and is considered to be a correlate of working memory consolidation [10]. In the 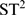 model, working memory encoding occurs by creating a binding link between types from stage one and tokens from stage two. Hence, the virtual P3 component contains activation from later parts of the first stage, the nodes in stage two and the binding link connecting the two stages.

#### Virtual ERPimages

Virtual ERPimages are used to qualitatively compare single trial dynamics of the model to human EEG data. They depict the individual simulated trials that underlie the average virtual ERP, just as human ERPimages depict the experimental trials that contribute to the grand average ERP. Further, the conditions plotted are defined exactly the same as with human ERPimages: the targets seen outside the AB condition includes trials with seen T2s at lag 8 following a seen T1, and the targets seen inside the AB condition includes trials with seen T2s at lag 3 following a seen T1.

In order to generate virtual ERPimages comparable to their human counterparts, we combined simulated trials across multiple complete runs of the model. For each of the 18 subjects in the human data, a complete simulation run of the 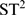 model was executed once, over all combinations of T1 and T2 strengths. For all trials in each such run, a small delay (fixed per subject) was introduced in the processing of stimuli presented to the model. This delay was a positive or a negative integer value, randomly sampled once per run, from a normal distribution with a mean of 0 ms and a standard deviation of 50 ms. It was ensured that, despite the introduction of this delay, the behavioural performance of the model was the same as that published in [5]. To generate the virtual ERPimage for a particular experimental condition, the corresponding trials from all the runs were collected together, sorted by 50% area latency within the 200–1100 ms window in each trial, and plotted as a colour-map with a vertical smoothing window of 15 trials to improve visual clarity.
